# A Case of Carcinoma of the Œsophagus

**Published:** 1908-06-06

**Authors:** Leonard A. Bidwell

**Affiliations:** Surgeon to West London Hospital and Dean of the College. A Clinical Lecture delivered at the Post-Graduate College, West London Hospital.


### Hospital Clinics.

### A CASE OF CARCINOMA OF THE ŒSOPHAGUS.

By LEONARD A. BIDWELL, F.R.C.S., Surgeon to West London Hospital and Dean of the College.

A Clinical Lecture delivered at the Post-Graduate College, West London Hospital.

The patient is a man aged 67, who has experienced some difficulty in swallowing for nearly two years, but it is only since Christmas 1906 that he has been unable to swallow solids; he has been very much worse since April 1907, and has emaciated rapidly during the last two months. Speech has been considerably affected for one month. When he first came as an out-patient I passed a bougie and discovered a stricture at the commencement of the œsophagus just behind the cricoid cartilage. A large bougie was afterwards passed with little difficulty. After this the patient was able to swallow fluids and semi-solids easily. A bougie was then passed twice a week, and the patient put on flesh; but during my absence from London for a month no bougie was passed, and on my return it was impossible to get any instrument through the stricture, whereupon he was admitted. He was then very emaciated, and had great difficulty in swallowing even a few drops of liquid, so the only chance of prolonging his life was to perform gastrostomy at once. This was done the same day, and four ounces of food was introduced into the stomach during the operation. The patient was fed, by a tube stitched into the stomach, every two hours for the first day and every four hours on the following day, four ounces being given in each feed on the first day, eight ounces on the second day, and one pint on the third day. The feed consisted of equal parts of beef-tea and peptonised milk with ℥ss, of brandy, and three eggs in the twenty-four hours. The operation gave great relief, and he was sitting up reading the paper on the second day. On the third day he became delirious, but became quieter after 40-grain doses of bromide of potassium had been given in his food. He is now on the ninth day quite quiet and sensible, the wound has healed well, but there is a little irritation round the fistula. His speech is now quite intelligible. He has certainly gained flesh, and is quite comfortable.

As carcinoma is not quite so common in the œsophagus as elsewhere, this case is worth a little consideration. In the first place, cancer of the œsophagus in my experience is much more common in males than in females; secondly, it is situated at the narrow parts of the œsophagus; and, thirdly, it does not produce marked symptoms for some time, and as the patient is usually past middle life the little difficulty in swallowing solids, which it at first causes, is attributed to dyspepsia.

Before considering the diagnosis and treatment, let me remind you of some points in the anatomy of the œsophagus. It is nine inches long and its termination is on the left side of the middle line, opposite to the body of the tenth dorsal vertebra; this on the back corresponds to the level of the tip of the seventh or eighth dorsal spine. Its commencement is opposite the body of the sixth cervical vertebra. It lies on the vertebral column above, and does not get close to the ribs till within about one inch of its termination. Where it lies on the ribs the deglutition sounds are best heard; this point is just to the left of the fifth or sixth dorsal vertebra or over the angle of the eighth rib. The diameter of a healthy œsophagus is about ¾-in., but on distension it increases to nearly 1½-in. There are three narrow parts in the œsophagus, where the diameter is nearly one quarter of an inch less than elsewhere; these are situated, one at its commencement, one 2¾-in. from the commencement (nearly opposite the sternal notch), and the last at the cardiac orifice of the stomach. It is at one of these narrow parts that carcinoma occurs. The other anatomical point that we must remember is that the œsophagus lies in close contact with the aorta, which accounts for the œsophageal obstruction caused by aneurysm, and that it has the vagi and recurrent laryngeal nerves on each side of it.

Now as regards diagnosis. It is rare for a case of cancer of the œsophagus to come under notice until serious impairment of swallowing has occurred, in fact until the patient can no longer swallow solids, and has even difficulty in swallowing liquids, and so has commenced to emaciate. The means of diagnosis consist of (1) the passage of a bougie, (2) auscultation, and (3) the examination of any vomited matter or detached piece of growth in the eye of a stomach tube. You must always remember that no bougie may be passed until the thorax has been carefully examined with a stethoscope to eliminate any question of aneurysm.

After auscultation a bougie may be passed, first to locate the exact distance of the stricture from the teeth, and secondly to estimate the amount of constriction present. My practice is to attempt to pass a full-sized instrument in the first instance, and then to introduce smaller ones till one passes through the stricture. Before passing the bougie, soften it well in hot water, and bend the tip slightly upwards so as to keep it clear of the larynx. It is best for the patient to sit and to keep his head in the natural position. Hold the bougie quite lightly in the right hand, and depress the tongue with one finger of the left hand; pass the bougie directly backwards till it touches the posterior wall of the pharynx, instruct the patient to swallow, and the bougie will glide on without any force being required. If the patient retches when the tube is introduced, apply cocaine to the pharynx. Never use force when passing a bougie.

Other methods of examination are the œsophagoscope and the $x$-rays. The œsophagoscope is rather unpleasant to the patient, and does not afford any greater information than the bougie. In using $x$-rays, the patient is asked to swallow some bismuth pills or some porridge containing bismuth. On examining his thorax with the fluorescent screen, the pills or porridge will be seen to be lodged just above the stricture.

Other symptoms met with in cancer of the œsophagus are (1) anæmia, (2) cachexia, and (3) enlargement of the cervical glands. The form of carcinoma most frequently met with in the œsophagus is a squamous epithelioma, and therefore the actual malignancy of the growth is slight. Scirrhus has been described, and in the neighbourhood of the stomach a cylindrical carcinoma may be found.

We may now consider the question of treatment. Owing to the relations of the œsophagus, complete removal of the growth is practically out of the question. Such an operation has been undertaken several times, but never with success. Treatment, therefore, is only palliative, and varies according to the position of the growth. Its object is to maintain a passage through the growth for as long as possible. In cases of carcinoma at the commencement of the œsophagus, I pass the largest possible bougie once or twice a week: this is done in all cases in which it does not cause much irritation or bleeding.

When the growth is situated behind the sternal notch or close to the cardiac end, the introduction and use of a Symond's tube will nearly always prove of value. In such a case, if I can get any bougie through the stricture I pass a Symond's tube of the same size as the bougie and leave it in for three or four days, or until it becomes blocked. I then withdraw and pass one of a larger size; the size is gradually increased till a No. 16 is passed, and this will require changing once every 10 or 14 days. The tubes are short, with a funnel-shaped top, to which are attached two silk threads; these are brought out of the mouth and fastened to the ear. Before introducing a Symond's tube the distance of the stricture from the teeth must be accurately measured on a bougie, and it is well to mark this distance on the introducer of the tubes; it will then be easy to ascertain when the tube has been properly passed. With a tube of small size liquids and custards can be swallowed with ease, and, with those of larger sizes, pounded meat and vegetables can also be taken. As a successful result of treatment by Symond's tube, I would refer to the following case. A gentleman aged 55 consulted me in July 1892; he was then extremely emaciated and feeble, and was on slop diet, which he had great difficulty in getting down. I commenced intubation immediately, and within a month I had introduced a full-sized tube. With this he was able to make hearty meals, and to a casual observer could eat anything. I dined with him at his club one night; after the soup, fish was brought in, and his portion looked exactly like a fillet of sole; it was, however, sole pounded with cream and then shaped to resemble a fillet. Then followed a lamb cutlet, which had also been first pounded, followed by game prepared in the same way, etc. In January, while getting over a stone fence out shooting he had a cerebral hæmorrhage, from which he died about 14 days later, which I consider a most merciful ending for an incurable malignant stricture. Since then I have treated many patients in this manner, and in the greater number with very great benefit. Occasionally I meet with a case in which the tube cannot be worn, and such I treat with bougies if they can be tolerated. When bougies cause pain I leave the patient till emaciation commences, and then immediately perform gastrostomy.

There are certain dangers connected with the use of Symond's tubes; they are said to cause ulceration within and abscesses outside the œsophagus, which may hasten death. These complications I have not seen, but I have had two cases in which the silk thread has been swallowed and then has blocked the lumen of the tube. I always impress on the patient the importance of watching the silk to see that it is not frayed by the teeth, and strongly urge them to come to me at once if one of the two threads becomes severed. The first case in which the silk threads were swallowed had been under treatment for three months, and had gained flesh, and was able to continue his work; he neglected my warning to come at once when one thread gave way, and a day or so later bit through and swallowed the second one. He was then unable to swallow anything, and as I was unable to reach the tube with œsophageal forceps, I did an œsophagotomy and removed the tube. In the second case it was suggested to me that I might get hold of the silk threads by using a coin catcher; this I did, and successfully caught the threads, but, unfortunately, in withdrawing the coin catcher the metal part of the instrument broke off from the whalebone and slipped back into the œsophagus. I then had an *x*-ray photograph taken, and the coin catcher was seen behind the second rib. I had the patient anæsthetised, and passed down a hollow bougie with a small guarded hook in its lumen; with this I succeeded in catching the thread of the tube, and in pulling on the thread brought the tube into the pharynx with the coin catcher in its funnel. At this moment the patient gave a violent expiratory effort, which dislodged the coin catcher from the tube, and the tube was ejected from the throat, but the coin catcher slipped back again into the œsophagus. Another *x*-ray photo was taken, and the coin catcher was seen to be in exactly the same position as before. As it could not be reached by forceps an œsophagotomy was performed, and the coin catcher removed. At the same time the stricture was thoroughly dilated with bougies through the œsophagotomy wound. The patient recovered perfectly, and required no further intubation for some months.

Though I am greatly in favour of intubation where it can be borne in preference to gastrostomy, yet in cases in which intubation cannot be tolerated I consider that one must not let the patient die of starvation, so gastrostomy should be performed before there is any real difficulty in swallowing a sufficient quantity of liquids. Time does not permit me to describe the various methods of performing gastrostomy, in all of which the chief aim is to prevent leakage of gastric juice through the fistula. The patient is fed at the time of the operation, and the tube remains *in situ* for nearly a fortnight, by which time the rest of the wound is healed. When the tube is first withdrawn there is some escape of gastric juice, but this soon decreases, and seldom gives any trouble. The irritation is best treated by dusting with bicarbonate of soda. The method of feeding has already been described. The prognosis in these cases is bad, although they regain strength for a little time after the operation; but few of my patients have lived longer than three months. I will not here describe the methods of treating inoperable cases by trypsin and the *x*-rays, as though I have tried the methods I have not seen any good result from their use.

